# Lean adoption, implementation, and outcomes in public hospitals: benchmarking the US and Italy health systems

**DOI:** 10.1186/s12913-022-07473-w

**Published:** 2022-01-29

**Authors:** Marta Marsilio, Martina Pisarra, Karl Rubio, Stephen Shortell

**Affiliations:** 1 Department of Economics, Management and Quantitative Methods (DEMM), Università degli Studi di Milano, Via Conservatorio, 7, 20122 Milan, Italy; 2 Center for Healthcare Organizational and Innovation Research, School of Public Health, University of California, Berkeley, Berkeley, California USA; 3 Center for Lean Engagement and Research in Healthcare, School of Public Health, University of California, Berkeley, Berkeley, California USA

**Keywords:** Lean healthcare, Public hospitals, Performance improvement, Outcomes, Benchmarking, Italian national healthcare system, United States healthcare system

## Abstract

**Background:**

Despite the growing interest in transformational performance improvement among nearly all countries, international benchmarking has rarely been used. Cross-comparative research could allow an appreciation of the extent of Lean’s use in healthcare and a better evaluation of possible cultural influences on Lean implementation. This study provides a comparative international benchmarking of Lean adoption, implementation, and outcomes of hospitals in the US and Italy.

**Methods:**

The National Survey of Lean, developed in 2017 in the US and adapted in Italy in 2019 was used to compare the two healthcare systems along three dimensions: the maturity of adoption, the implementation approach, identifying both strategic and operational activities and tools, and the Lean performance, investigated through patients, employed, and affiliated staff, costs, and service provision areas. Descriptive statistics including T-tests were used to examine differences between the two countries on the study variables.

**Results:**

Lean has been adopted less by Italian public hospitals (36%) than US public hospitals (53%). Each country averages 4 years of experience with Lean. Italian hospitals reported being at a higher maturity stage while the US implemented a more system-wide approach, developing Lean in more operational units. The daily management system, leadership commitment, education and training indexes were higher or the same in the US while in Italy, hospitals had a higher self-reported performance index.

**Conclusion:**

This exploratory work is one of the first international benchmarking studies on Lean implementation in healthcare systems using a standardized survey with a common set of definitions and questions. The study identifies different forms of Lean implementation that can be adopted, both at strategic and operational levels, with related perceived outcomes. Despite the US public hospitals being more likely to report a higher number of units using Lean, a higher daily management system index and use of Lean tools, Italian hospitals report more achievements primarily due to Lean. Further research can build on these findings by examining the relationship between Lean adoption/implementation and independent, objective performance measures.

## Background

Rising costs of care, budget constraints, aging population, chronic diseases and increasing patient expectations are forcing healthcare providers to adopt innovative approaches and methodologies in an effort to improve their performance [1, 2]. Among these practices, Lean management stands out in terms of diffusion in the health care management field [1, 3–7]. First introduced into healthcare in the early 1990s [8], Lean is defined as an overall management/operating system that uses a continuous improvement culture [1, 3, 4] and focuses on meeting customer needs (i.e. the needs of patients, internal staff, and the organization), improving quality by reducing waste (i.e. those activities that are not adding value) [9–11], optimizing organizational processes and patient flows, and creating value, with the direct involvement of the organization’s staff [12]. Lean involves a set of practices and tools to assess, improve, and monitor work processes [13].

Despite the growing interest in transformational performance improvement among nearly all countries [1], benchmarking has rarely been used at an international level, even though it could be useful to expand knowledge in the field [14]. Cross-comparative research could allow an appreciation of the extent of Lean’s use in healthcare and a better evaluation of possible cultural influences on Lean adoption and implementation [15].

To fill this gap, we conducted an international benchmarking comparison of Lean adoption, implementation and outcomes using data from a National Survey of Lean (NSL)/Transformational Performance Improvement (TPI) in hospitals in the United States (US) and Italy. The potential benefits of Lean management principles applied to hospitals have nurtured the debate among scholars and practitioners in both healthcare systems. In several systematic and bibliometric literature reviews [15–17] the US emerged as the leading country in terms of publications on Lean in healthcare. Italy is one of the UE countries where the attention to lean in health care organizations has been rapidly increasing [17–19]. Thus, these two countries could offer insight not only on the Lean initial adoption approach, but also on the implementation strategies and outcomes achieved.

These two healthcare systems differ in major respects. The Italian National Healthcare System (INHS) is a public universal system, providing free care to all people. It follows the principles of the Beveridge System, in which resources are collected by general taxation at central level and then devolved to Regional Healthcare Services in charge of the provisioning of services [20]. In contrast, the US Healthcare System (USHS) is a mixed private/public system. People aged 65 and above are provided health insurance coverage by the Federal Medicare tax-supported program while most of the remaining are covered through employer-sponsored private health insurance coverage, or those below poverty by a mixed public/private Medicaid program. Despite reforms initiated by the Affordable Care Act (ACA) of 2010 about 30 million Americans or about 7% of the population remain uninsured due to gaps in the available coverage programs.

Despite these context-specific differences, public hospitals are crucial and critical providers in both healthcare systems. In Italy they account for 80% of public health expenditures, representing 73% of total health expenditure (Italian Ministry of Health 2020); in the US, comparable data are not available but public hospitals constitute about 22% (approximately 1000 hospitals) of all US hospitals providing care to about 14% of all inpatients.

Some common factors influencing healthcare Lean transformation may be operating in public hospitals [21, 22]. These include competing or even contradictory political, regulatory, and commissioning priorities [13] in which heavy bureaucracy, rigid policies, and regulations often reduce flexibility and complicate Lean implementation [23]. Thus, despite the differences in the overall organization of the Italian and US health systems, focusing on the implementation of Lean TPI in public hospitals in the two countries may provide some important lessons.

## Methods

### Study design

Lean management scholars call for the use of reliable methods to measure leanness (a global metric to assess lean production implementation) [24], to capture the dynamic interpretation of lean in different contexts [25]. Healthcare production processes are characterized by a series of processes that inevitably influence the Lean management approach. Accordingly, Lean healthcare has been developing into a major strand of research since the early 2000s [15]. The healthcare NSL survey was developed in 2017 based on literature review, discussion with Lean experts, and pilot testing with 12 US Lean performance improvement specialists.

The NSL represents a valid and pragmatic instrument that allows benchmarking among different national health systems [4].

The survey was approved by the Institutional Review Board of the University of California, Berkeley. The 20-min online survey was administered in the US between May and September 2017.

In 2019 the survey was adapted to the Italian national context and pilot tested with seven Lean performance improvement specialists and sent to all public healthcare organizations. The 20-min online survey was administered in Italy between January and June 2019.

The surveys were completed by the chief transformation officer, chief performance improvement officer, chief quality officer, or equivalent position title in each hospital in both countries.

### Measures

Accordingly to the NSL work [1], the major topics covered by the survey included the adoption and implementation of Lean or related performance improvement system by the hospital; date of initial adoption; extent of current use; Lean implementation approach; self-reported maturity in using Lean; hospital Lean strategies; use of a central improvement Lean team; extent of the daily management system; recourse to Lean tools; Lean diffusion within units; use of external consultants; the presence of a True North vision; the finance, human resources (HR), and information technology (IT) departments’ involvement; education and training; and self-reported performance impacts primarily attributable to Lean.

Scholars agree that Lean is an organization-wide socio-technical performance improvement system [3, 18]. Thus, the actual degree of implementation throughout the organization as opposed to mere adoption is more likely to be associated with positive hospital performance [26].

This paper provides a theoretical framework based on three main dimensions aiming to investigate the relationship of a system-wide approach to the adoption and implementation of Lean with its achievements. These include:

- the *maturity of adoption* (concerning how much Lean management is diffused within hospital units, the timing since it has been implemented by the hospital and Lean self-reported maturity);
- the *implementation approach* (concerning the methodologies, principles and techniques used, the people involved and the operations mechanisms), identifying both *strategic* and *operational* activities and tools; and
- the Lean performance, investigated through 15 self-reported achievements grouped in four main areas [14]: *patients, employed and affiliated staff, costs and service provision*.

Table 1 summarizes the specific items investigated in the comparison between the two healthcare systems.

**Table 1:** Surveys’ dimensions and variables

Dimensions	Variables
Adoption maturity	• Number of years doing Lean • Lean Maturity Assessment • Number of units doing Lean
Strategic implementation approach	• Approach for Lean adoption • Lean leadership commitment index • Central improvement team • External consultant
Operational implementation approach	• Daily management system index • Index support by HR, IT and Finance units • Reward and Recognition • Lean team composition and leadership • Main tools used • Education and training index • Staff Involvement index
Performance	• Self-reported index • Self-reported impact on patients (Improved patient satisfaction scores, Reduced medical errors, Reduced one or more types of hospital-acquired infections, Reduced hospital readmissions within 30 days of discharge, Reduced risk adjusted 30-day mortality, Reduced ambulatory care sensitive admissions) • Self-reported impact on employed and affiliated staff (Improved employee engagement in their work, Reduced employee turnover) • Self-reported impacts on costs (Reduced expenditures in two or more departments, eliminated waste in two or more processes or departments, Reduced average length of stay) • Self-reported impacts on service provision (Increased throughput in the emergency department, Increased throughput in the operating rooms, Increased throughput in the cardiac care unit, Increased throughput in med/surg nursing units)

The indexes have been measured as follows [1, 6]:

- The *leadership commitment index* was built with the following eight-items: how clear leaders communicate the reason(s) for implementing Lean, the expected outcomes, employee’s time and resources investment in Lean, successful projects with teachings about Lean, use of benchmarks to assess progress, leaders’ provision of needed resources, team champions/sponsors identification, and the recourse to a patient-centered care. The choice was from “strongly disagree” to “strongly agree” for each item. Agree and strongly agree answers were put together and one point to each of the eight items was given (range 0 to 8). The Cronbach alpha reliability coefficient was 0.70 for Italy and 0.81 for US.
- The *daily management* system (DMS) *index* consisted of nine-items with the aim of investigating whether or not managers were involved in few activities or used Lean tools (i.e., daily huddles, “gemba” walks, visual management, analysis tools such as scatter plots, A3 thinking, teaching Lean methods/tools, standard work, value stream mapping, and Plan-Do- Study-Act (PDSA) cycles). Points were collected for each “YES” of the items with a range from 0 to 9. Cronbach’s alpha reliability coefficient was 0.73 for Italy and 0.75 for US*.*
- The *HR, IT and Finance indexes* were measured by three composite indexes. HR index included five items - if HR: helps with Lean goals, has the role of advisor for managers, provides managers the data and analysis needed, works with leaders in redefining job roles and responsibilities or if working with Lean is considered in the recruitment process. The Cronbach’s reliability coefficient was for Italy 0.82 and 0.74 for the US. The finance index included three items - if the finance department: helps with Lean goals, contributes providing managers the data and analysis needed, has the role of advisor for managers. The Cronbach’s alpha reliability coefficient was 0.6 for Italy and 0.66 for the US. The IT function included six items - if the IT department: helps with Lean goals, has the role of advisor for managers, provides managers the data and analysis needed, the hospital has ready access to integrated data of clinical and operational processes, managers have very timely and accurate data. The Cronbach’s alpha for Italy was 0.78 and 0.80 for the US. For each item, the response categories were “strongly disagree”, “disagree”, “neither agree nor disagree”, “agree”, and “strongly agree”. The index was built with the number of responses “agree” or “strongly agree”.
- The *education and training index* aimed at measuring the degree of Lean education and training of the hospital’s staff (i.e., managers, nurses, and physicians) with training in scientific approaches to problem solving choosing between the following percentage ranges: 0% (categories of 0), 1–24% (category 1), 25–49% (category 2), 50–74% (category 3), and 75–100% (category 4). The averaged across the three groups—managers, nurses, and physicians— was conducted and an average score from 0 to 4 was built. The Cronbach’s alpha reliability coefficient was 0.88 for Italy and 0.82 for US.
- The *staff involvement index* aimed at measuring the involvement of managers, doctors, and nurses in the use of tools and activities for Lean implementation: establishing goals for Lean improvement; using value stream mapping/mapping of value flow, fishbone diagrams, A3 Reports, fast improvement events (Rapid Improvement Events - RIE) or related tools and approaches; coaching activities; attending daily huddles; establishing processes that help sustain improvements. Each “YES” was given 1 point. The scale ranges from 0 to 5.
- The *self-reported performance index* was built assigning 1 point to each “YES” response to the 15 performance improvement areas that could be primarily attributed to Lean implementation. The scale ranges from 0 to 15. The Cronbach’s alpha reliability coefficient was 0.84 for Italy and 0.89 for the US.

### Data analysis

We used descriptive statistics with means and standard deviations (SD) to present the findings on each variable and summary scales of interest. In addition, an independent two-sample t-test to determine significant differences in implementation and achievements of Lean between the US and Italy. All analyses were conducted using R.

## Results

### The sample

Table 2 compares the respondents and non respondents in both countries by hospital bedsize. The survey was administered in the US to 672 public acute hospitals with a 30% completion rate (n = 282 hospitals). In Italy the survey was sent to 198 public acute hospitals with a 46% completion rate (n = 91 hospitals). In both countries the respondents were from somewhat larger institutions than the non-respondents. Due to a policy of supporting mergers to create larger size hospitals, the Italian hospitals had an average bedsize of 873 in comparison to the much smaller average bedsize of 131 for the US public hospitals. Other than the differences in size the respondents in both countries were generally similar to all public hospitals in each country.

**Table 2:** Comparison of Responders and Non-responders on bed size (Public Hospitals)

	US				Italy			
	Non-respondents (*N* = 672)		Respondents (*N* = 282)		Non-respondents (*N* = 107)		Respondents (*N* = 91)	
	*N*	%	*N*	%	*N*	%	*N*	%
**Public hospitals**	672	70	282	30	107	54	91	46
**Bed Size**	(*N* = 672)		(*N* = 282)		(*N* = 101)		(*N* = 89)	
Small	504	75	190	67.4	34	33.6	24	27
Medium	128	19.1	58	20.6	61	60.5	54	61
Large	40	6	34	12.1	6	5.9	11	12
Mean	106		131		738		873	

### Lean diffusion and adoption

As shown in Table 3, Lean diffusion is considerably higher in the US hospitals than in the Italian hospitals (respectively 53 and 36%). Nonetheless, the average number of years doing Lean, 4 years, is similar in both countries. However, in Italy, hospitals reported that Lean was more expanding to other units and being mature than in the US even though US public hospitals reported twice as many units doing Lean as in Italy.

**Table 3:** Lean adoption maturity

	US				Italy			
	N	%	Mean	SD	N	%	Mean	SD
**Current Performance Improvement Approach**								
	282				97			
Benchmarking for Best Practices	131	46			44	45		
Lean	149	53			35	36		
The Model for Improvement	54	19			NA	NA		
High Reliability Organization (HRO)	81	29			69	71		
Value-based Healthcare	NA	NA			9	9		
FOCUS-PDCA	88	31			4	4		
Six Sigma without Lean	18	6			1	1		
**Currently doing any Lean?**	282				97			
Yes	149	53			35	36		
No	133	47			62	64		
**MATURITY ADOPTION**								
**Number of years doing Lean**	143		4.6	3.56	35		4	3.57
**Lean self- reported maturity**	144				34			
Still in the new start-up stage	32	22			10	29		
Beyond start-up, but challenged moving forward	49	34			4	12		
Expanding to other units and getting traction	55	38			16	47		
Mature transformational performance improvement	8	6			4	12		
** Number of units doing Lean**	138		11.9	7.69	35		6	3.87

### Strategic implementation approach

As shown in Table 4, the use of a hospital-wide approach to Lean implementation is more developed in the US compared to Italy where there is a preference for a small number or even single department applications. The majority in both health systems, however, started with a model cell and with approximately half reporting a “True North vision” reflecting overall hospital strategic goals and priorities. As shown, only 6% of the Italian hospitals called for an expert outside the organization for assistance in implementing Lean while this is a common practice in the US hospitals (72%).

**Table 4:** The strategic implementation approach

	US				Italy			
	N	%	Mean	SD	N	%	Mean	SD
**Approach at the beginning of Lean implementation**	142				34			
Some elements hospital-wide	52	37			3	9		
Some elements in a small number of departments	52	37			22	65		
Some elements in a single department	13	9			9	26		
Comprehensive DMS hospital-wide	11	8			0	0		
Comprehensive DMS in a small number of departments	9	6			0	0		
Comprehensive DMS in a single department	5	4			0	0		
**Initiated Lean with a model cell**	143				35			
Yes	84	59			24	69		
No	59	41			11	31		
**Have a True North vision**	139				35			
Yes	68	49			19	54		
No	71	51			16	46		
**Overall Lean leadership commitment index (range: 0–8)**	139		4.7	2.48	35		4.6	2.18
**Have a central improvement team**	139				35			
Yes	87	63			20	57		
No	52	37			15	43		
**Ever used an outside consultant**	138				35			
Yes	100	72			2	6		
No	38	28			33	94		

### Operational implementation approach

As shown in Table 5, the DMS index score is twice as high in the US than in Italy (4.8 out of 9 versus 2.4 out of 9). The level of staff (managers, doctors, and nurses) involvement is less for Italy (1.04) than for the US (3.9), highlighting that in the Italian context the engagement of nurses, doctors, and managers in Lean tools and activities is low. Generally, the US hospitals reported a higher percentage of rewards and recognitions from departments and external organizations, while in Italy rewards and recognition operate mainly at the hospital level. In Italy, only 65% of the respondents reported the use of any reward and recognition system, mainly at the hospital level (57%).

**Table 5:** The operational implementation approach

	US				Italy			
	N	%	Mean	SD	N	%	Mean	SD
**Daily management system index (range: 0–9)**	135		4.8	2.53	35		2.4	1.55
**HR index (range: 0–5)**	129		2.3	1.82	35		2.3	1.85
**IT index (range: 0–6)**	129		2.7	2.05	35		3.8	1.97
**Finance index (range: 0–3)**	130		2.0	1.07	35		1.8	1.11
**Lean team multi-professionalism**	68				14			
Information Technology	32	47			7	50		
Human Resources	16	24			4	29		
Finance	20	29			3	21		
**Staff involvement index (range: 0–6)**	76		3.9	1.34	35		1.04	1.41
**Education and training index (range: 0–4)**	128		1.79	0.89	35		1.7	0.93
**Reward and Recognition**	293				23			
Departments	103	35			4	17		
External Organizations	84	29			6	26		
Hospital	106	36			13	57		
**Number of tools reported as high or very high use (range: 0–14)**	130		4.3	3.6	35		3.3	3.3

Table 6 provides a comparison of Lean tools and methods between the two countries. Tools and techniques are fundamental in the Lean implementation process. In the US hospitals there is a statistically significant higher adoption for just-in-time (JIT) process or inventory management, Kaizen improvement events, and training in process improvement tools for employees. There are no statistically significant differences in the use of other tools or methods.

**Table 6:** Comparison on Lean tools and methods adoption

Tools and Methods	US (1)		Italy (2)		t-test difference
	N	Mean [SD]	N	Mean [SD]	(1)–(2)
5 s: redesign of physical workspace	129	3.977 [1.320]	35	4.143 [1.115]	−0.166
A3 thinking	129	3.341 [1.355]	35	3.114 [1.255]	0.227
Analysis tools such as scatter plots, Pareto charts	129	3.705 [1.208]	35	3.800 [1.256]	−0.095
Daily huddles	129	4.628 [1.409]	35	4.171 [1.014]	0.456
Just-in-time process or inventory management	129	3.891 [1.427]	35	2.743 [1.221]	1.149***
Kaizen improvement events	129	3.318 [1.566]	35	2.143 [1.264]	1.175***
Mistake-proofing	129	3.318 [1.256]	35	2.886 [1.367]	0.432
Redesign for continuous flow [pull system, etc.]	130	3.469 [1.325]	35	3.571 [1.290]	−0.102
PDSA	129	4.388 [1.239]	35	4.171 [1.014]	0.216
Six Sigma DMAIC methodology	130	3.115 [1.622]	35	2.971 [1.382]	0.144
Standard work	130	3.923 [1.198]	35	4.086 [1.040]	−0.163
Training in process improvement tools for employees	129	3.310 [1.535]	35	2.714 [1.467]	0.596*
Value stream process mapping	130	3.477 [1.342]	35	3.629 [1.215]	−0.152
Visual management such as huddle boards	128	4.070 [1.421]	35	3.743 [1.245]	0.327

The value displayed for t-tests are the differences in the means across the groups

***, **, and * indicate significance at the .1, 1, and 5% critical level respectively

### Lean outcomes

The self-reported outcomes primarily due to Lean implementation are indicated in Table 7. The Italian hospitals report a higher score for patient satisfaction, hospital readmissions within 30 days of discharge, risk adjusted 30-day mortality, reduced length of stay and increased throughput (in the operating rooms, in the cardiac care unit and in medical/surgical nursing units) than the US.

**Table 7:** Comparison on Lean self-reported performance

Variable	US (1)		Italy (2)		t-test difference
	N	Mean [SD]	N	Mean [SD]	(1)–(2)
**Self-reported performance index (range: 0–15)**	127	7.1 [3.7]	35	8.6 [3.8]	
**PATIENT**					
Improved patient satisfaction scores	110	0.727 [0.447]	31	0.968 [0.180]	−0.240^**^
Reduced medical errors	104	0.702 [0.460]	25	0.880 [0.332]	−0.178
Reduced one or more types of hosp-acquired infections	100	0.640 [0.482]	20	0.700 [0.470]	−0.060
Reduced hospital re-admissions within 30 days of discharge	103	0.524 [0.502]	22	0.773 [0.429]	−0.248^*^
Reduced risk adjusted 30-day mortality	83	0.253 [0.437]	16	0.688 [0.479]	−0.434^﻿***^
Reduced ambulatory care sensitive admissions	68	0.279 [0.452]	17	0.529 [0.514]	−0.250
**EMPLOYED AND AFFILIATED STAFF**					
Improved employee engagement in their work	114	0.816 [0.389]	32	0.938 [0.246]	−0.122
Reduced employee turnover	84	0.310 [0.465]	14	0.500 [0.519]	−0.190
**COSTS**					
Reduced expenditures in two or more departments	112	0.795 [0.406]	24	0.792 [0.415]	0.003
Eliminated waste in two or more processes or depts	121	0.926 [0.263]	33	0.970 [0.174]	−0.044
Reduced average length of stay	102	0.461 [0.501]	30	0.800 [0.407]	−0.339^***^
**SERVICE PROVISION**					
Increased throughput in the emergency department	114	0.737 [0.442]	28	0.679 [0.476]	0.058
Increased throughput in the operating rooms	103	0.544 [0.501]	30	0.900 [0.305]	−0.356^***^
Increased throughput in the cardiac care unit	91	0.275 [0.449]	23	0.696 [0.470]	−0.421^***^
Increased throughput in med/surg nursing units	100	0.510 [0.502]	27	0.815 [0.396]	−0.305^**^

The value displayed for t-tests are the differences in the means across the groups

^***﻿^, ^**^, and ^﻿*^ indicate significance at the .1, 1, and 5% critical level respectively

## Discussion

Although Lean by itself has been adopted somewhat less by Italian public hospitals than US public hospitals, each country averages 4 years of experience with Lean indicating it is a somewhat new approach to improving hospital performance among public hospitals in each country.

Italian hospitals, however, consider their experience more mature despite having only half of the number of units involved in Lean projects compared to the US. This could be due to the fact that in Italy there is quite a diffuse adoption of other related more traditional performance improvement approaches, such as High Reliability Organization and benchmarking, that are more widely spread among the Italian hospitals.

Considering the strategic implementation approach, the majority in both countries started with a model cell and with around half reporting a True North vision. Nonetheless, the US hospitals show a higher adoption of a system-wide approach, while in Italy, hospitals prefer “a project at a time”, with a small number of departments involved and a very scarce use of outside consultants. The adoption of an innovative performance approach such as Lean compared to other more diffused approaches in the Italian system may benefit, at least in the first stage of its adoption, from the knowledge and expertise developed by outside expert consultants. Moreover, Lean literature considers leadership commitment to be a fundamental driver for Lean implementation success and sustainability over time [3]. The study reveals that Leadership commitment, is an area requiring further attention in both countries given the relatively low average score (4.5 out of 8) on this index. Thus, a longitudinal analysis could help to investigate if this weakness could constrain the future ability of hospitals to maintain the same level of maturity adoption and performance.

On the operational implementation approach, the results show a higher score for the US hospitals in the use of a daily management system. The INHS score is particularly low (2.4 over 9), reflecting an insufficient familiarity with common Lean management activities.

The level of staff (managers, doctors, and nurses) involvement is also significantly lower in Italy (Italy 1 vs US 4). Lean scholars call for a high level of involvement for all the staff as a driver to assure a successful implementation [12, 15, 27]. The weakness of staff involvement in the Italian context could be troubling considering the staff education and training index is also low. US hospitals also report a low staff education and training score, but with a higher level of staff involvement and greater use of more Lean tools such as JIT inventory, Kaizen, and training process improvement methods.

Regarding performance, Italian hospitals perceive greater impact primarily of Lean implementation on patients, costs, and service improvement.

Considering the relatively weaker Italian scores in the strategic and operational domain compared to the US, future research should focus on examining the impact through independent objective hospital performance measures. The self-reported scores may reflect more “wish fulfillment” than what might actually be the case. Additionally, defining objective measures of Lean hospital performance improvement across an array of financial, patient outcome, and patient satisfaction indicators could help the hospital leaders to be more aware of what “adoption of Lean” actually means in their hospital, helping the implementation process [4].

There is growing interest in cross-national comparative benchmarking studies of Lean [14, 28] that also recognize differences in each countries healthcare systems. This work is among the first to meet this need. Further research should define objective measures for the four performance areas identified in this paper (patient, employed and affiliated staff, cost-financial, service provision) and investigate their applicability and relevance in different health systems [4].

Our findings need to be considered within the context of study limitations. First, the survey was completed by a single informant, identified by the hospital as the most knowledgeable person to respond. However, other leaders or staff in the organization may have responded differently. Second, the findings are limited by common instrument bias, in that the questions about performance impact, although widely separated in the question ordering, were asked on the same questionnaire as the other measures. Third, the self-reported performance data should be viewed with caution. As previously indicated, future research should link the study variables to independent, objective measures of hospital performance such as risk-adjusted mortality, hospital readmissions within 30 days, and risk-adjusted inpatient expenses per discharge [26]. Fourth, moving from this exploratory work, larger samples of hospitals are needed to conduct multivariate analysis of study variables to assess the independent effect of the study variables when comparing healthcare systems. Further, ongoing administration of the NSL will facilitate the examination of potential casual effects of the study variables.

## Conclusion

This work is one of the first international benchmarking studies on Lean adoption, implementation and outcomes in healthcare systems using a standardized survey with a common set of definitions and questions.

The theoretical framework used to classify the items of the surveys has been developed to identify the indicators that can be used to measure the *maturity of adoption* and the *strategic* and *operational implementation approach*, thus identifying a standardized frame to analyze the diffusion of Lean in a healthcare system. The framework also identifies four main areas (*patients, employed and affiliated staff, costs and service provision)* of performance outcomes to compare the leanness between different healthcare systems.

Finally, this framework supports examining the relationship between Lean adoption/implementation and performance outcomes.

Despite a more system-wide implementation approach among US public hospitals, their efforts seem to be associated with lower perceived performance outcomes primarily due to Lean than hospitals in Italy. There is need to examine Lean using independent, objective performance measures. A growing evidence base regarding Lean and related transformational performance initiatives will assist policymakers and professionals in all countries in making more informed decisions on improving public sector hospital performance.

## Data Availability

The datasets generated during the current study are not publicly available due to confidentiality of some of the data but are available from the corresponding author on reasonable request.

## Abbreviations

NSL: National Survey of Lean
TPI: Transformational Performance Improvement
INHS: Italian National Healthcare System
SD: Standard deviations
JIT: Just-in-time
US: United States
USHS: US Healthcare System
ACA: Affordable Care Act
HR: Human resources
IT: Information technology
DMS: Daily management system
PDSA: Plan-Do- Study-Act
RIE: Rapid Improvement Events
HRO: High Reliability Organization
